# Combined physical and cognitive training in a community aging center serving a socioeconomically vulnerable population: a feasibility study

**DOI:** 10.1186/s11556-026-00420-2

**Published:** 2026-06-16

**Authors:** Yoconda Arias, Briceida Bergado, Jorge A Bergado, Cindy Carriazo, José Chaar, Shadye Matar, Leonilde Morelo, Efraín Mora, Randolph Nudo, Oscar Vargas

**Affiliations:** 1 Universidad del Sinú, Montería, Colombia; 2 Department of Physical Medicine and Rehabilitation, University of Kansas Medical Center, Kansas, USA

**Keywords:** Older adults, Community-based intervention, Physical exercise, Cognitive training, Feasibility study, Functional performance

## Abstract

**Background:**

Structured physical activity programs are increasingly promoted to maintain functional capacity in older adults; however, evidence regarding their implementation in real-world community settings, particularly in socioeconomically vulnerable populations, remains limited. Multimodal interventions integrating physical and cognitive components may provide additional benefits, yet feasibility data from resource-constrained environments are scarce. This single-arm feasibility study evaluated the feasibility, safety, and adherence of a combined physical and cognitive training program delivered in a community aging center serving a socioeconomically vulnerable population with exploratory assessment of functional and cognitive outcomes.

**Methods:**

In this single-arm feasibility study, 50 community-dwelling adults aged > 55 years attending a community aging center serving a socioeconomically vulnerable population were enrolled following medical, physical, and neuropsychological screening; 46 completed the intervention and post-intervention assessment. Participants attended one-hour sessions conducted on alternate days for four months, alternating between structured physical exercise and supervised cognitive activities. Primary feasibility outcomes included recruitment, retention, adherence, and safety. Secondary exploratory outcomes included functional capacity assessed by the Six-Minute Walk Test (6MWT) and cognitive performance assessed using the abbreviated NEUROPSI battery. Within-participant changes were analyzed descriptively and inferentially.

**Results:**

Of 50 enrolled participants, 46 (92%) completed the intervention and post-intervention assessment. Mean attendance was 18.7 ± 9.3 h (range 3–39). No adverse events attributable to the intervention were reported. Mean 6MWT distance increased from baseline to post-intervention by 140 m (Cohen’s d = 1.58, *p* < 0.001), while oxygen saturation and blood pressure responses remained stable. Improvements in walking distance were moderately associated with session attendance. Modest increases were also observed in global cognitive performance at post-intervention. Given the single-arm design and interval between baseline and intervention, outcome findings should be interpreted as exploratory.

**Conclusions:**

A structured combined physical and cognitive training program was feasible and safe in a community aging center serving a socioeconomically vulnerable population, with high retention and no intervention-related adverse events. Improvements in functional capacity were observed, with exploratory evidence of cognitive change. These findings support the practicality of implementing supervised multimodal programs in real-world community environments and provide a foundation for future controlled studies.

**Supplementary Information:**

The online version contains supplementary material available at 10.1186/s11556-026-00420-2.

## Background

Age-related declines in physical function contribute substantially to loss of independence and reduced quality of life. Regular physical activity has been consistently associated with preservation of functional capacity and mobility across the aging spectrum [1]. Interventions targeting strength, balance, and aerobic capacity improve objective measures of physical performance [1] and are increasingly recognized as important components of healthy aging [2].

Beyond its effects on physical performance, accumulating evidence indicates that exercise may also influence cognitive function through vascular, metabolic, and neuroplastic mechanisms [2–5]. Structured cognitive engagement has independently demonstrated benefits in memory and executive domains [6, 7]. Consequently, multimodal interventions integrating physical and cognitive components have been proposed as potentially synergistic strategies, with large-scale trials such as FINGER reporting favorable outcomes in selected populations [8–11].

Despite encouraging findings from controlled trials, few studies have examined the feasibility, adherence, and safety of structured multimodal programs delivered in real-world, resource-constrained community settings. Evidence from Latin American and Caribbean populations remains particularly limited, especially within socioeconomically vulnerable day-care centers [12]. Careful characterization of implementation challenges, participation variability, and preliminary functional responses in these environments is therefore necessary before larger controlled trials can be designed and adequately powered.

Based on these considerations, we developed a structured combined physical and cognitive training program adapted for delivery within a community aging center serving a socioeconomically vulnerable population. The present study aimed to evaluate the feasibility, safety, and adherence of this program in that setting, with exploratory assessment of functional and cognitive measures in a single-arm design.

## Methods

### Study design, setting, and timeline

This study was designed as a single-arm feasibility study evaluating the implementation of a combined physical and cognitive training program in a real-world community aging center. As a single-arm feasibility study without randomization or a control group, it was not prospectively registered as a clinical trial. The primary objective was to assess implementation, safety, and adherence rather than to test efficacy. Accordingly, no formal a priori power calculation was performed. The sample size was determined pragmatically based on center capacity, available resources, and staffing constraints.

The intervention was delivered at Centro de Vida “Espíritu de Dios” in Montería, Colombia, which serves older adults from a socioeconomically vulnerable population.

Baseline assessments were conducted between November 20 and December 15, 2023. Due to temporary administrative closure of the center, the intervention commenced on August 12, 2024, and concluded on November 23, 2024. No structured exercise or cognitive training program was delivered at the center during the closure period, and no interim study-related assessments were performed. Post-intervention assessments, including the second 6-minute walk test and neuropsychological evaluation, were performed between November 25 and December 13, 2024. The study is reported in accordance with the CONSORT extension for pilot and feasibility trials (See Supplementary File 1).

### Participants

Community-dwelling adults aged over 55 years who were registered beneficiaries of the center were screened for participation. The lower age threshold of 55 years was selected pragmatically to align with the population served by the participating community aging center and to capture individuals entering the period of increased risk for age-related functional and cognitive decline. All participants provided written informed consent prior to enrollment. Individuals with severe cognitive impairment, inability to ambulate independently, or medical conditions that precluded safe participation in moderate physical activity were excluded based on predefined criteria. A concise summary of inclusion and exclusion criteria is presented here; full criteria are provided in Supplementary File 2.

All volunteers received detailed information about the study procedures and provided written informed consent in accordance with established ethical guidelines for research in humans [13]. The study was approved by the Bioethics Committee of Universidad del Sinú (Act 003, April 18, 2023) and by the administrative authorities of the center.

From the eligible cohort, a subsample of participants was randomly selected for inclusion in the intervention based on infrastructure and staffing constraints. Selection was not stratified or balanced according to sex, education, or other covariates.

Participant selection occurred in three steps:

1. Assessment of eligibility based on predefined inclusion and exclusion criteria.
2. Medical, physical, and cognitive evaluation.
3. Baseline measurement of study variables.

### Feasibility and safety outcomes

Primary feasibility outcomes included recruitment, retention, session attendance rates, adherence to the planned program, and occurrence of adverse events. Health-related events during the intervention period were recorded and evaluated for potential association with program activities.

### Eligibility assessment and baseline evaluation

To confirm eligibility, standardized instruments were administered as follows:

- Barthel Index: assessment of basic activities of daily living [14].
- Lawton-Brody Scale: evaluation of instrumental activities of daily living [15].
- Abbreviated Montreal Cognitive Assessment (MoCA): administered according to validated procedures for low-education populations [16]. Scores were interpreted using education-adjusted norms as recommended in the validation guidelines.
- 15-item Yesavage Geriatric Depression Scale: evaluation of depressive symptoms [17, 18].

All scales were administered in the morning in a quiet setting by trained students under the supervision of a geriatric specialist.

### Exploratory functional and cognitive measures

#### Six-Minute Walk Test (6MWT)

Functional capacity was assessed using the Six-Minute Walk Test [19]. The primary functional measure was the distance walked in six minutes. The test was conducted according to established guidelines. Blood pressure, oxygen saturation, and perceived exertion were measured before and after testing using standard procedures. Predicted normal walking distance was calculated according to established reference equations [20].

#### Neuropsychological assessment

Cognitive performance was assessed using the short version of the NEUROPSI battery [21]. The instrument evaluates orientation, attention, executive function, memory, language, visuoconstructional abilities, and calculation. Scores were adjusted for age and education according to manual guidelines. Assessments were administered by trained psychology and medical students under professional supervision.

#### Intervention

The intervention consisted of alternating physical and cognitive training sessions conducted in the morning over a four-month period. Sessions were delivered on alternate days, with each session lasting approximately 60 min. A total of 35 physical and 35 cognitive sessions were completed. All sessions were conducted in small groups under supervision to ensure safety and engagement.

Each participant could attend up to 70 total sessions (35 physical and 35 cognitive) over the intervention period. Because attendance was voluntary and scheduling was flexible, participation was recorded in total hours rather than discrete session counts. No a priori adherence threshold was defined, and adherence was assessed descriptively relative to the planned program.

The components of the physical and cognitive training programs were informed by prior literature on multicomponent exercise and cognitive stimulation interventions in older adults and were adapted to the resources and population of the participating center [22, 23].

#### Physical training

Physical training sessions were delivered in small groups by advanced physiotherapy students under supervision of experienced specialists. Each session lasted approximately 60 min and was structured to target multiple functional domains, including agility, muscular strength, balance, coordination, proprioception, and spatial awareness.

Sessions typically included a combination of the following components: (1) dynamic mobility and agility exercises (e.g., walking between cones, multidirectional stepping, and rhythm-based movement sequences); (2) strength training using body weight, light external loads, or elastic resistance bands to target both upper and lower extremities; (3) balance and proprioceptive exercises performed on stable and unstable surfaces, including tasks requiring postural control and weight shifting; and (4) coordination and spatial orientation tasks involving object manipulation (e.g., ball handling) and navigation in different directions.

Exercises were adapted to the functional capacity of participants and progressed in complexity or intensity based on individual tolerance and performance. Rest periods were incorporated as needed to maintain safety and participation. All sessions were conducted in small groups to allow close supervision and minimize fall risk. Attendance and tolerance were recorded at each session.

#### Cognitive training

Cognitive training sessions were conducted on alternating days with physical training sessions and were delivered by psychology and medical trainees under professional supervision. Each session lasted approximately 60 min and was conducted in small groups.

The cognitive intervention followed a multicomponent approach targeting key domains relevant to age-related cognitive decline, including memory, attention, executive function, language, and functional independence. Session activities included: (1) memory exercises such as word and object recall, repetition tasks, and recognition activities; (2) executive function training through sequencing, planning, and problem-solving tasks; (3) language and communication exercises including verbal fluency, reading comprehension, and guided discussion; and ([4) attention and processing tasks requiring sustained focus and mental flexibility.

Additional components included training in activities of daily living and cognitive compensation strategies, such as the use of written reminders, calendars, and structured routines to support functional independence. Fine motor and coordination tasks were incorporated using manual activities to reinforce cognitive-motor integration. Group-based exercises, including cooperative tasks and guided social interaction, were used to promote communication, engagement, and social participation.

To support adherence, a simple behavioral reinforcement strategy was implemented. Attendance was tracked and displayed as a non-monetary group-based approach to promote engagement, and participants received non-monetary recognition based on participation. Periodic psychoeducational sessions were also provided to reinforce understanding of age-related cognitive changes and to encourage continued engagement in cognitive and physical activity outside of the program.

### Statistical analysis

Data were summarized using descriptive statistics (means and standard deviations for continuous variables; frequencies and percentages for categorical variables). Within-participant changes from baseline to post-intervention assessment were evaluated using paired t-tests. One-way ANOVA was used to explore associations between cognitive performance and categorical variables such as education level and MoCA score categories. Effect sizes (Cohen’s d for standardized mean change) were calculated for primary exploratory comparisons. Statistical significance was set at *p* < 0.05, and exact p-values are reported where applicable. Physiological responses to the 6MWT were summarized descriptively to characterize safety and expected responses to exertion and were not included in the prespecified inferential analyses. Missing post-intervention data were not imputed. Given the single-arm feasibility design, all inferential analyses were considered exploratory.

## Results

Given the single-arm feasibility design, all functional and cognitive outcomes are reported as exploratory and should not be interpreted as evidence of efficacy.

### Participant flow and baseline characteristics

**Fig. 1:**
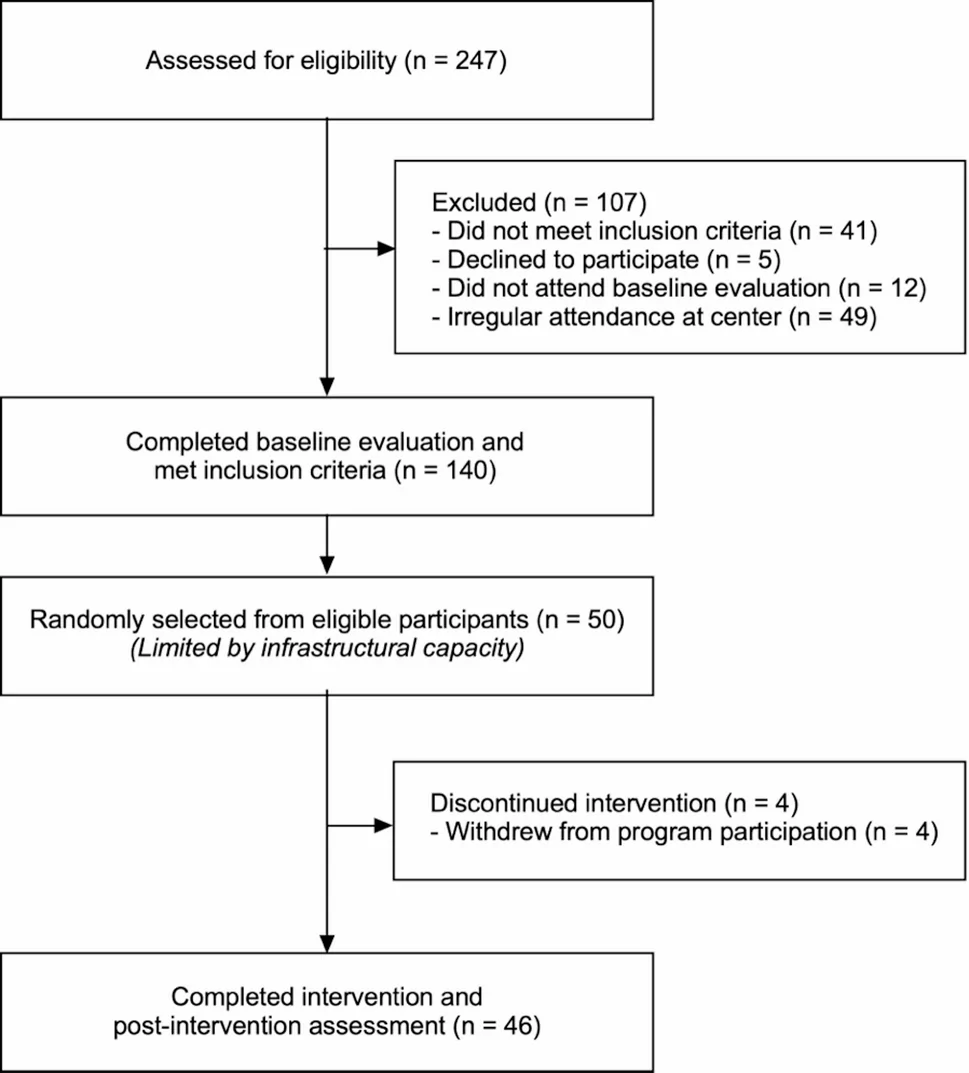
Flow of participants from eligibility assessment through intervention completion and post-intervention evaluation

A total of 247 beneficiaries were assessed for eligibility. Of these, 41 did not meet inclusion criteria, five declined participation, 12 did not attend the scheduled evaluation, and 49 were excluded because of irregular attendance at the center. The remaining 140 beneficiaries met inclusion criteria and completed baseline evaluation. Due to infrastructural and staffing constraints, 50 participants were randomly selected from the eligible cohort to participate in the intervention. Selection was not stratified or balanced according to sex, education, or other covariates. Four subsequently discontinued participation, resulting in 46 participants completing the intervention and post-intervention assessment (Fig. 1). Reasons for discontinuation were not systematically recorded, as participation was voluntary and follow-up of non-completers was limited in this community-based setting.

Baseline demographic and clinical characteristics of the final analytic sample (*n* = 46) are presented in Table 1. The mean age was 67 years, and 33 participants (71.7%) were women. Arterial hypertension was the most common chronic condition. Baseline functional, cognitive, and mood measures are summarized in Table 2. Mean Barthel and Lawton-Brody scores were close to their respective maximum values, and the mean 15-item Geriatric Depression score was 1.6 (SD 1.6). The mean abbreviated MoCA score (12-point version) was 7.3 (SD 2.2).

**Table 1:** Baseline demographic and clinical characteristics (*n* = 46)

Characteristic	Value
Age, years – mean (SD)	67 (5.3)
Sex, n (%)	
Female	33 (71.7%)
Male	13 (28.3%)
Education level, n (%)	
Illiterate	4 (8.7%)
Incomplete elementary	13 (28.3%)
Completed elementary	10 (21.7%)
Incomplete secondary	8 (17.4%)
Completed secondary	10 (21.7%)
University/technical degree	2 (4.3%)
Cohabitation status, n (%)	
Living with son/daughter	18 (39.1%)
Living with partner	16 (34.8%)
Living with other relatives	2 (4.3%)
Living with non-relatives	1 (2.2%)
Living alone	9 (19.6%)
Chronic conditions, n (%)^a^	
No chronic condition	2 (4.3%)
Arterial hypertension	16 (34.8%)
Diabetes mellitus	1 (2.2%)
Respiratory disease	3 (6.5%)
Hypertension + diabetes mellitus	11 (23.9%)
Diabetes mellitus + dyslipidemia	1 (2.2%)
Diabetes mellitus + other	1 (2.2%)
Dyslipidemia + other	2 (4.3%)
Other chronic conditions	9 (19.6%)

^a^ Categories represent mutually exclusive clinical groupings as recorded in the clinical files

**Table 2:** Baseline functional, cognitive, and mood characteristics of the study sample

Measure	*n*	Mean (SD)	Scale maximum
Barthel Index (functionality)	46	96.0 (2.0)	100^a^
Lawton-Brody Scale (independence)	46	7.8 (0.9)	8^a^
MoCA test (cognition)	43^d^	7.3 (2.2)	12^b^
Yesavage Depression Scale (mood)	46	1.6 (1.6)	15^c^

^a^Higher scores indicate greater functional independence

^b^MoCA scores are based on the abbreviated 12-point MoCA version validated for low-education populations [16]

^c^The 15-item GDS was used [17]; lower scores indicate better mood (scores *≤* 4 = no depressive symptoms)

^d^MoCA data were available for 43 participants due to incomplete baseline testing in three individuals

### Feasibility, adherence and safety

**Fig. 2:**
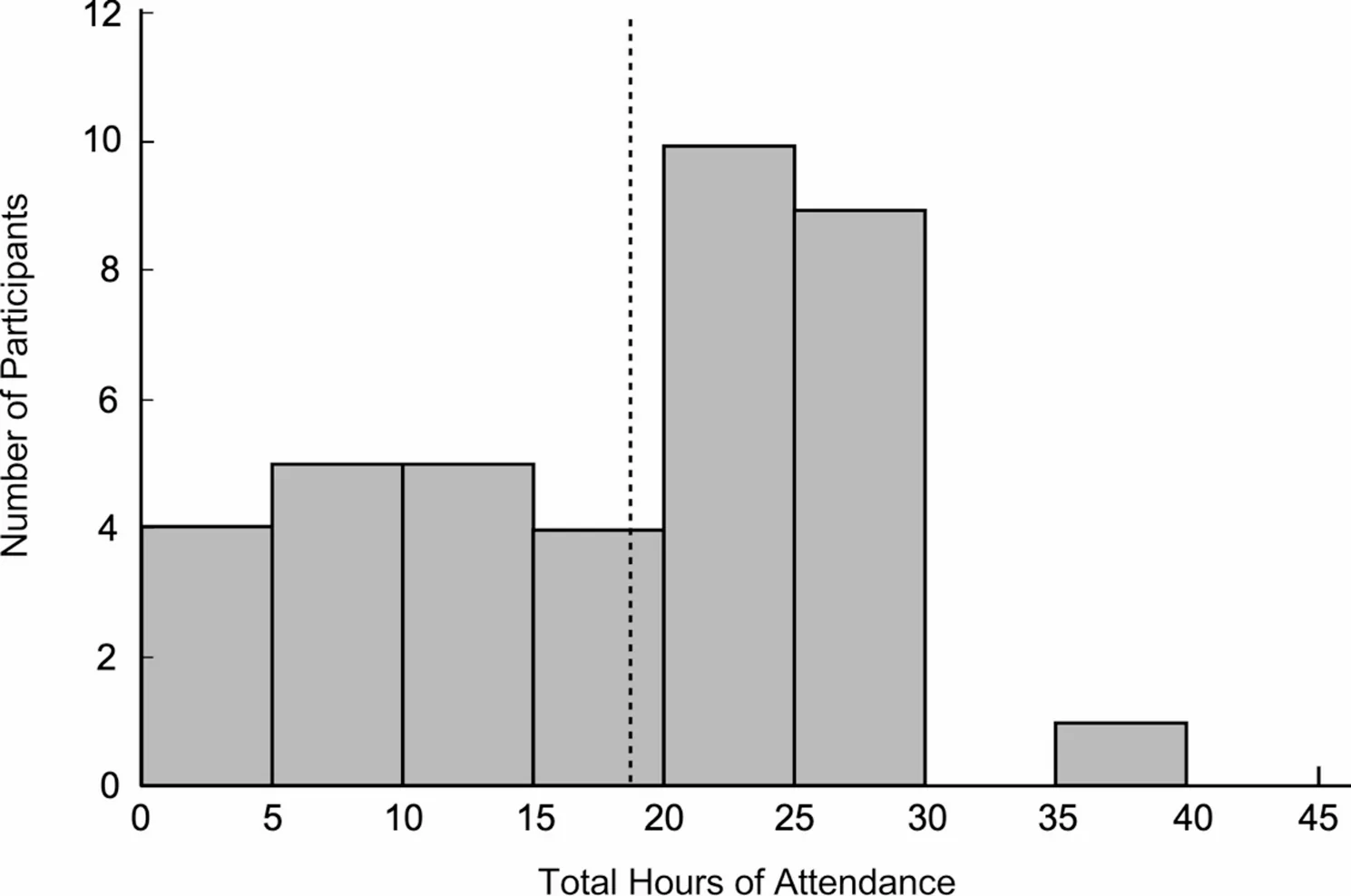
Distribution of total hours of attendance during the intervention (*n* = 46). The dashed line indicates mean attendance (18.7 h)

Of the 50 individuals selected for the intervention, 46 (92%) completed the program and post-intervention assessment. Attendance varied substantially, with a mean total participation of 18.7 ± 9.3 h (range 3–39). Approximately half attended fewer than 20 h of training. Participation was voluntary, and specific reasons for absences were not systematically recorded. Consistent with the feasibility design, adherence was evaluated descriptively, and no predefined threshold for adequate participation was applied. The distribution of attendance is shown in Fig. 2.

No adverse events, including falls or syncope, occurred during supervised training sessions. Four participants (8.7%) experienced medical events during the study period; all were adjudicated by clinical staff as unrelated or unlikely related to the intervention (Table 3). No intervention-related adverse events were identified.

**Table 3:** Medical events reported during the intervention

Participants (*n*)	Event	Relation to intervention	Outcome
1	Catheterization for venous obstruction	Unlikely related	Reduced attendance
2	Planned ocular surgery	Not related	Reduced attendance
1	Venous thrombosis (leg)	Unlikely related	Reduced attendance

Events occurred among 46 participants enrolled in the intervention. Four participants (8.7%) experienced medical events during the study period. All events were adjudicated by clinical staff as unrelated or unlikely related to study activities. No intervention-related adverse events were identified

### Exploratory functional outcomes

At baseline, participants walked 488 ± 58 m, corresponding to 98.9% of age- and sex-predicted values. Following the intervention period, mean 6MWT distance was 629 ± 112 m, representing a mean within-participant change of 140 m (paired t-test, *p* < 0.001; *n* = 46). The standardized mean change (Cohen’s d) was 1.58. Change in walking distance was moderately correlated with total hours of attendance (*r* = 0.45, *p* = 0.01; Supplementary Figure S1).

Physiologic responses to the 6MWT were comparable at baseline and post-intervention assessments. Systolic and diastolic blood pressure, heart rate, and perceived exertion increased from pre- to post-test at both time points in a pattern consistent with expected physiological responses to exertion, while oxygen saturation remained stable (Table 4). No consistent differences were observed between baseline and post-intervention assessments.

**Table 4:** Functional circulatory responses during the 6-minute walk test at baseline and post-intervention

Physiologic measures during the 6-minute walk test	Baseline		Post-intervention	
Variable	Pre	Post	Pre	Post
Systolic blood pressure (mmHg)	125 ± 18	137 ± 24	120 ± 12	145 ± 29
Diastolic blood pressure (mmHg)	74 ± 9	77 ± 9	68 ± 6	71 ± 6
Heart rate (beats/min)	82 ± 12	101 ± 21	80 ± 10	102 ± 15
Borg scale	1.0 ± 0.0	4.3 ± 2.3	1.0 ± 0.0	4.7 ± 2.9
SPO_2_ (%)	96.3 ± 4	96.6 ± 3	98.0 ± 2	98.4 ± 1

Values represent participants with complete baseline and post-intervention assessments (n = 46). Within each time point, post-test values were higher than pre-test values for systolic and diastolic blood pressure, heart rate, and perceived exertion, while SpO2 (%), remained stable. No conistent differences were observed between baseline and post-intervention evaluations. Values are mean ± SD

### Exploratory cognitive outcomes

Mean NEUROPSI total score was 74.9 ± 15.9 at baseline and 80.1 ± 18.0 at post-intervention, corresponding to a mean within-participant change of 5.1 points (paired t-test, *p* = 0.038; *n* = 46). The standardized mean change (Cohen’s d) was 1.19. Domain-level scores at baseline and post-intervention are presented in (Table 5).

**Table 5:** Domain-specific NEUROPSI performance at baseline and post-intervention (*n* = 46)

Cognitive Domain	Subdomain	Max Score	Mean ± SD Baseline	% of Max	Mean ± SD Post-intervention	% of Max
Orientation	Time and place	6	5.7 (0.6)	95	5.6 (0.8)	93
Attention & Concentration	Digits (reverse)	6	2.5 (1.2)	42	2.5 (1.2)	42
	Visual detection	16	7.2 (4.5)	45	8.1 (4.4)	51
	Serial subtraction (20–3)	5	2.8 (1.9)	56	2.9 (1.8)	58
Memory	Spontaneous recall	6	3.9 (0.9)	65	4.0 (0.9)	67
	Visuospatial processing	12	8.1 (2.8)	68	8.5 (3.1)	71
Language	Naming	8	7.6 (0.5)	95	7.8 (0.5)	98
	Repetition	4	3.9 (0.6)	98	4.0 (0.2)	100
	Comprehension	6	3.9 (1.3)	65	4.3 (1.4)	72
	Semantic fluency	4	2.0 (0.5)	50	2.3 (0.6)	58
	Phonologic fluency	4	1.5 (0.7)	38	1.4 (0.6)	35
Reading/Writing	Reading comprehension	3	0.8 (1.1)	27	1.0 (1.1)	33
	Dictation	2	1.2 (1.0)	60	1.2 (1.0)	60
Executive Functions	Similarities	6	2.8 (2.2)	47	3.9 (2.1)	65
	Calculation	3	1.4 (1.1)	47	1.4 (1.1)	47
	Sequencing	1	0.1 (0.3)	10	0.1 (0.3)	10
	Changing hand position	4	2.0 (1.3)	50	2.2 (1.4)	55
	Alternating hands	2	1.1 (0.8)	55	1.2 (0.8)	60
	Opposite reactions	2	1.3 (0.6)	65	1.2 (0.8)	60
Evocation	Visuospatial memory	12	6.2 (2.8)	52	6.7 (3.3)	56
	Spontaneous verbal memory	6	1.8 (1.6)	30	2.3 (1.8)	38
	Memory keys	6	2.2 (1.4)	37	2.2 (1.8)	37
	Recognition	6	5.2 (1.1)	87	5.0 (1.4)	83
Total		130	74.9 (15.9)	58.9	80.1 (18.0)	61.5

Values represent participants with complete baseline and post-intervention assessments (*n* = 46). For total NEUROPSI score, Post-intervention values were significantly higher than baseline (paired t-test, *p* < 0.038; Cohen’s d = 1.19)

In exploratory analyses, total NEUROPSI scores were associated with educational level (F₅,₃₉ = 6.35, *p* < 0.001) and MoCA score categories (F₉,₃₂ = 4.30, *p* < 0.001).

The exploratory association between attendance and change in walking distance is shown in Supplementary Figure S1.

## Discussion

In this single-arm feasibility study conducted in a community-based day-care center, a combined physical and cognitive training program was feasible, safe, and associated with exploratory improvements in walking capacity and modest cognitive gains. Baseline assessments were performed several months before initiation of the intervention due to administrative closure of the center. Although no structured exercise or cognitive training was provided during this interval, unmeasured changes in functional or cognitive status may have occurred. This temporal separation should be considered when interpreting post-intervention findings. While similar multicomponent physical and cognitive interventions have been examined in prior studies, the present work contributes by evaluating the feasibility, safety, and real-world implementation within a community aging center serving a socioeconomically vulnerable population. This setting provides practical insight into recruitment, adherence variability, and delivery under resource-constrained conditions that are less frequently captured in controlled trials.

This intervention was conducted in a specialized day-care center serving older adults in Montería, Colombia. The recruited cohort reflected the characteristics of individuals typically attending similar community-based programs in the region, including representation across varied socioeconomic backgrounds. The predominance of women is consistent with demographic patterns observed in older adult community programs and may reflect broader socioeconomic and lifespan employment disparities affecting women in this setting.

The prevalence and pattern of chronic conditions were typical for older adults in Colombia, with arterial hypertension being the most common diagnosis, followed by metabolic and respiratory diseases [24–26]. Despite these comorbidities, participants demonstrated preserved independence in activities of daily living, as reflected by high Barthel and Lawton-Brody scores [27, 28].

Cognitive screening with the abbreviated MoCA yielded relatively low mean scores. Educational attainment likely contributed to this pattern, consistent with prior Colombian cohorts demonstrating strong associations between MoCA performance and years of schooling [29]. These findings suggest that lower scores should be interpreted within educational and cultural context rather than evidence of pathological cognitive decline alone. Consideration of education-adjusted norms is particularly important when applying standardized cognitive instruments in low- and middle-income settings.

Affective status, assessed with the 15-item Yesavage Geriatric Depression Scale (GDS) [17], was largely within the normal range. Only one participant met criteria consistent with severe depressive symptoms, indicating low depressive symptom burden in this cohort. This prevalence was lower than that reported in some larger community-based studies of older adults in Colombia [30]. The structured and socially interactive nature of the day-care environment may be relevant, although this was not formally examined in the present study.

Baseline physical performance, evaluated with the 6MWT, indicated preserved functional capacity for age. The mean walking distance of 488 m corresponded to 98.9% of the age- and sex-predicted value according to Enright’s equation. Physiologic responses were appropriate, with expected increases in blood pressure and heart rate and stable oxygen saturation during exertion. These findings indicate that, despite common comorbidities, overall functional performance in this cohort was largely preserved [24, 25, 27]. The relatively high baseline performance should be considered when interpreting post-intervention changes and may limit generalizability to frailer populations.

At baseline, neuropsychological assessment demonstrated lower performance in attention, concentration, memory processing, and executive function, domains commonly influenced by normal cognitive aging [28–30]. Educational level was associated with overall test performance, consistent with the well-established role of education in shaping cognitive reserve across the lifespan.

Overall, the cohort demonstrated preserved functional independence and cognitive performance within the context of common chronic conditions and socioeconomic constraints. These characteristics support the ecological validity of evaluating a structured combined training program within this community-based setting.

Post-intervention assessments included repeat administration of the 6MWT and the abbreviated NEUROPSI battery. Walking distance increased in nearly all participants, and the magnitude of improvement was positively correlated with the number of training sessions attended (Supplementary Figure S1), consistent with a potential exposure-response relationship. The standardized mean change was large (Cohen’s d = 1.58), and the absolute increase is in a range that exceeds commonly reported minimal clinically important differences for the 6MWT in older adults. However, in the absence of a control group, regression to the mean, motivational influences, and test familiarity cannot be excluded, and causal inference is not possible. Variability in attendance is consistent with voluntary participation in community-based settings and provides practical insight for future implementation and trial design.

A substantial body of human and animal research demonstrates that regular physical exercise influences brain structure and function across the lifespan [1–5, 22, 31–34]. Reported mechanisms include enhanced cerebral perfusion, structural adaptations in regions such as the hippocampus, and upregulation of neurotrophic factors that support neuroplasticity [3, 35]. Although the present study did not assess mechanistic outcomes, this literature provides biological plausibility for the observed exploratory changes in cognitive performance following combined physical and cognitive engagement.

Cognitive training programs have demonstrated benefits for memory, attention, and executive function in both healthy aging and mild-to-moderate dementia [6, 7, 36]. Intervention effects are frequently moderated by educational level, consistent with the role of cognitive reserve in shaping individual responsiveness. Although the relative contributions of physical and cognitive training remain under investigation [11], randomized trials have suggested that combined multimodal approaches may produce broader or more sustained benefits than single-domain interventions in some populations [8]. These findings provide a conceptual framework for understanding how physical activity and cognitive engagement might interact. Integrative models of neurorehabilitation similarly emphasize the value of combining physical and cognitive stimulation to support adaptive plasticity [37]. However, the present single-arm feasibility design does not permit evaluation of comparative efficacy or mechanistic interaction.

As with the improvement observed in the 6MWT, the modest cognitive gains detected on the NEUROPSI may partly reflect test-retest familiarity. Practice effects are common in repeated cognitive assessment and cannot be excluded. The four-month duration of the intervention may be insufficient to demonstrate clinically meaningful changes in cognitive function, particularly in the absence of a control group and given the potential influence of practice effects in repeated neuropsychological testing.

The present feasibility study provides several practical considerations for the design of a future controlled trial in which functional and cognitive outcomes would be specified as primary endpoints. Elements that should be retained include the community-based delivery model, supervised small-group sessions, and the combined physical and cognitive structure, all of which were feasible and safe in this setting. However, a future trial should incorporate a concurrent control group, prospectively define adherence targets, record attendance at the session level in addition to total hours, systematically document reasons for missed participation, and include participant-reported acceptability or satisfaction measures. Because baseline and intervention phases in the present study were separated by an administrative closure, future studies should also minimize delays between baseline assessment and intervention onset. If cognitive outcomes are to be primary endpoints, a longer intervention duration and a prespecified assessment schedule may also be warranted.

We did not perform a formal sample size calculation for the present study because its primary purpose was feasibility. Although the observed changes in functional and cognitive measures may inform preliminary planning, effect sizes derived from a small single-arm study are likely to be unstable and may overestimate the true intervention effect. A future sample size calculation should therefore be based on the primary endpoint selected for the definitive trial, the clinically meaningful difference to be detected, and preferably estimates derived from controlled data.

The relatively young mean age of the cohort should also be considered when interpreting the findings. Participants demonstrated relatively preserved baseline functional capacity, which may have contributed to the magnitude of observed improvements and could limit generalizability to older or more frail populations. It is also important to note that, in this setting, attendance at the community aging center was not restricted to individuals with significant comorbidity but instead reflected access to social and health-support services in a vulnerable population. As such, the study population may differ from more clinically impaired cohorts typically included in rehabilitation trials.

### Strengths and limitations

This study has several strengths. It was conducted in a real-world community setting within a public day-care center serving older adults from varied socioeconomic backgrounds, enhancing ecological validity. The intervention integrated structured physical and cognitive components over an extended period under supervised conditions, allowing systematic monitoring of adherence and safety. Standardized and validated instruments were used to assess functional, cognitive, and mood outcomes.

Several limitations warrant consideration. The sample size was modest, and participants were recruited from a single institution, limiting generalizability. The single-arm design precludes causal attribution of observed changes. Variability in attendance reflects the realities of voluntary participation in community-based settings, and practice effects in repeated cognitive assessments cannot be excluded. The interval between baseline assessment and initiation of the intervention represents an additional methodological limitation, as interim changes were not monitored. Participant-reported satisfaction or acceptability of the intervention was not systematically assessed, limiting interpretation of engagement and perceived benefit. Finally, participants demonstrated relatively preserved baseline functional capacity, which may limit applicability to frailer populations. These considerations are inherent to early-stage feasibility studies and underscore the need for larger, controlled trials.

## Conclusion

Older adults attending specialized day-care centers represent a practical and ecologically valid population for evaluating interventions aimed at preserving physical and cognitive function during aging. In this single-arm feasibility study, a structured combined physical and cognitive training program was well tolerated and associated with observed improvements in walking capacity and modest exploratory changes in cognitive performance, despite variability in adherence. These findings support the feasibility of implementing supervised multidomain programs in resource-constrained community settings. Larger, multicenter controlled trials are required to confirm efficacy and identify predictors of individual responsiveness.

## Supplementary information

Supplementary Material 1.

Supplementary Material 2.

Supplementary Material 3.

## Data Availability

The datasets generated and analyzed during the current study are not publicly available due to the inclusion of sensitive health and cognitive data from a small community-based sample but are available from the authors on reasonable request, subject to institutional and ethical approval.

## Abbreviations

6MWT: Six-Minute Walk Test
MoCA: Montreal Cognitive Assessment
GDS: Geriatric Depression Scale
NEUROPSI: Neuropsychological Battery in Spanish
MCI: Mild Cognitive Impairment
SD: Standard Deviation
ANOVA: Analysis of Variance
SpO₂: Peripheral oxygen saturation
CI: Confidence interval
