# Supplementary material for: Combined physical and cognitive training in a community aging center serving a socioeconomically vulnerable population: a feasibility study
Source: Eur Rev Aging Phys Act. 2026 Jun 16;23:32. doi: 10.1186/s11556-026-00420-2 (PMC13501603; doi:10.1186/s11556-026-00420-2)
Supplement: Supplementary file 2 [file 11556_2026_420_MOESM2_ESM.docx]

**Appendix 1. Inclusion and exclusion criteria**

Inclusion

- Being a registered beneficiary of the participating community aging center
- Signed informed consent to participate.
- Age > 55 years.

Exclusion criteria

- Unable to ambulate independently (including use of cane or similar assistive device).
- Neurologic disease affecting movement (e.g., stroke, multiple sclerosis, Parkinson’s disease).
- High-risk clinical conditions, including:
  - Uncontrolled atrial or ventricular arrhythmia
  - Aortic aneurysm
  - Severe aortic stenosis
  - Acute endocarditis or pericarditis
  - Uncontrolled arterial hypertension (> 180/100 mmHg)
  - Acute thromboembolism
  - Acute or severe cardiac insufficiency
  - Acute or severe respiratory insufficiency
  - Uncontrolled symptomatic orthostatic hypotension
  - Uncontrolled diabetes mellitus
  - Recent bone fracture
  - Recent myocardial infarction (within the previous 6 months) or unstable angina
- Any other health condition considered of risk by the physician in charge.
