# Supplementary material for: Combined physical and cognitive training in a community aging center serving a socioeconomically vulnerable population: a feasibility study
Source: Eur Rev Aging Phys Act. 2026 Jun 16;23:32. doi: 10.1186/s11556-026-00420-2 (PMC13501603; doi:10.1186/s11556-026-00420-2)
Supplement: Supplementary file 3 [file 11556_2026_420_MOESM3_ESM.docx]

Supplementary Figure S1. Association between total hours of attendance and change in 6-minute walk test distance (n = 46). The solid line represents the fitted linear regression; shaded area indicates the 95% confidence interval.
